# The first crystal structure of a family 45 glycoside hydrolase from a brown‐rot fungus, *Gloeophyllum trabeum* GtCel45A

**DOI:** 10.1002/2211-5463.13774

**Published:** 2024-02-04

**Authors:** Laura Okmane, Louise Fitkin, Mats Sandgren, Jerry Ståhlberg

**Affiliations:** ^1^ Department of Molecular Sciences Swedish University of Agricultural Sciences Uppsala Sweden

**Keywords:** Cel45A, cellulase, cellulose, endoglucanase, glycoside hydrolase GH45

## Abstract

Here we describe the first crystal structure of a beta‐1,4‐endoglucanase from a brown‐rot fungus, *Gloeophyllum trabeum Gt*Cel45A, which belongs to subfamily C of glycoside hydrolase family 45 (GH45). *Gt*Cel45A is ~ 18 kDa in size and the crystal structure contains 179 amino acids. The structure is refined at 1.30 Å resolution and *R*_free_ 0.18. The enzyme consists of a single catalytic module folded into a six‐stranded double‐psi beta‐barrel domain surrounded by long loops. *Gt*Cel45A is very similar in sequence (82% identity) and structure to *Pc*Cel45A from the white‐rot fungus *Phanerochaete chrysosporium*. Surprisingly though, initial hydrolysis of barley beta‐glucan was almost twice as fast in *Gt*Cel45A as compared to *Pc*Cel45A.

## Graphical abstract

We described the crystal structure of a GH45 subfamily C member from a brown‐rot fungus, compared it to a nearly identical subfamily C member from a white‐rot fungus, made sequence comparisons, and created consensus sequences for all GH45 subfamilies to highlight strictly conserved residues. Additionally, enzymatic degradation rate of barley betaglucan was compared between the two subfamily C members.

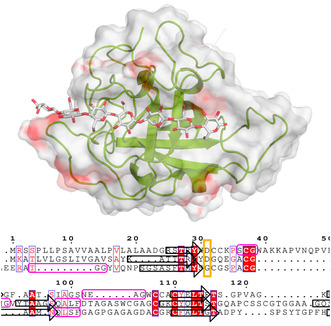

**Abbreviations**
CBM: carbohydrate binding module
GH45: glycoside hydrolase family 45
LPMO: lytic polysaccharide monooxygenase
RMSD: root mean square deviation

Brown rot fungi are major wood decomposers in coniferous forests. They are considered generalists or gymnosperm specialists in regard to host preference [1]. As the name suggests, a visual consequence of the degradation is browning of residual wood. That is because brown rot fungi primarily depolymerize cellulose and hemicellulose, and leave most of the lignin in the residues [2, 3]. Substrate degradation is achieved by secretion of various enzymes, such as cellulases, LPMOs and xylanases [4, 5, 6]. In comparison to white rot fungi, brown rot fungi contain less glycoside hydrolase and LPMO encoding genes, yet glycoside hydrolase family 45 (GH45) subfamily C genes appear to be more common in brown rot fungi [7]. The brown rot fungus *Gloeophyllum trabeum* is known to secrete all types of the aforementioned enzymes [8, 9].

Fungal cellulases and their use as tools in utilizing industrial lignocellulosic waste have been a research topic for more than 60 years. Perhaps the most studied is the application of fungal cellulases to generate biofuels from cellulosic biomass, often carried out by introducing potent fungal cellulases in the enzyme mixtures. However, a rapid and complete cellulose degradation is not always the desired characteristic in an industrial cellulase. For example, for cellulases used in washing powders, mild cellulolytic activity is desirable to avoid bulk degradation of intact cotton fibers. A common cellulase used for this purpose is the GH45 cellulase from *Humicola insolens*, HiCel45A [10, 11, 12, 13].

GH45 enzymes are generally small and have the smallest catalytic modules among GH families. Although often called cellulases, they commonly have broad specificity and hydrolyze other cell wall polysaccharides more readily (e.g. beta‐glucan, glucomannan), with low activity on insoluble cellulose [10, 14]. Currently, ~ 570 entries are listed in GH45 in the CAZy database, ~ 40 from bacteria, the great majority from fungi, and fewer from other eukaryotes (e.g. mollusks, insects, crustaceans, nematodes).

Phylogenetic analyses have divided GH45 family into three subfamilies. The subfamilies are referred to as either A, B, and C or according to a CAZy database classification as 1, 2, and 3, respectively [10, 15, 16]. The division of GH45 in subfamilies A, B, C predates the CAZy division. To this date, subfamily A, which includes HiCel45A, has been the most studied and subfamily C the least. Subfamilies B and C are more similar than subfamily A, to each other and to non‐hydrolytic cell wall‐active proteins called expansins, loosenins, and swollenins [17]. All these proteins contain a conserved double‐psi beta‐barrel domain (DPBB), also known as the GH45‐like domain. Loosenins and most GH45s consist of this domain alone, while expansins and swollenins also contain a beta‐sandwich domain (CBM63) in combination with the DPBB. Swollenins and some GH45s (e.g. HiCel45A) have an additional carbohydrate‐binding module attached with a flexible linker. There are also examples of GH45s with more complex domain architectures.

GH45 enzymes from all three subfamilies employ an inverting hydrolysis mechanism, with an aspartate residue acting as catalytic acid, which is also conserved in expansins, loosenins and swollenins [14]. However, the Asp proposed to act as catalytic base in subfamilies A and B, is missing in subfamily C, as well as in the non‐hydrolytic proteins [15]. Instead, an asparagine at another position has been proposed to act as catalytic base in GH45 subfamily C, based on neutron diffraction structure studies of *Pc*Cel45A from the white‐rot fungus *Phanerochaete chrysosporium* [18]. Although the mechanism of subfamily C may differ from A and B, subfamilies B and C are more alike in regard to reaction product profile [14].

Crystal structures of nine GH45 enzymes are available in the PDB (rcsb.org), six from kingdom Fungi (ascomycetes and basidiomycetes) and three from Metazoa (gastropod, bivalve, springtail), but no structure from brown rot fungi. There are structures for six members of subfamily A, two members of B (from mollusks) and only one of subfamily C, *Pc*Cel45A. Here we describe a second structure of a GH45 subfamily C enzyme—*Gt*Cel45A from the brown‐rot fungus *Gloeophyllum trabeum*.

Recombinant expression of *Gt*Cel45A, biochemical characterization and activity profiling have been published previously, in comparison with a subfamily A enzyme, MtGH45 from the ascomycete *Myceliopthora thermophila* [19]. Activity optima for *Gt*Cel45A were at pH 5 and 65 °C, and activities were in a similar range to MtGH45 on carboxymethyl cellulose, beta‐glucan, lichenan, and Avicel substrates.

## Materials and methods

### Cultivation, expression, and purification

Spores of *Aspergillus nidulans* A773 strain expressing *Gt*Cel45A (GenBank: EPQ56593) were kindly provided by Dr. Fernando Segato, University of Sao Paulo, Brazil. The generation of constructions is described by Berto *et al*. [19]. Approximate protein size is 18.4 kDa (183 aa), the calculated pI is 4.5.

A spore suspension in water was made from a 13‐day old sporulating culture growing on potato dextrose agar (PDA) plates. To prepare a pre‐culture, 50 mL of minimal medium (70 mm NaNO_3_, 7 mm KCl, 6 mm KH_2_PO_4_, 6 mm K_2_HPO_4_, 4.3 mm MgSO_4_, 20 μm FeSO_4_·7 H_2_O, 80 μm ZnSO_4_·7 H_2_O) supplemented with pyridoxine (1 mg·L^−1^) and maltose (3%) were inoculated with 200 μL of the spore suspension. The pre‐culture was incubated in shake flasks at 25 °C, 120 rpm for 10 days.

Protein expression was done in the same medium at 30 °C in 1 L shake flasks, 4 × 350 mL cultures, shaking at 75 rpm. Cultures were harvested after 7 days, twice filtered (through 1 μm GF/B Whatman glass‐fiber filter, followed by vacuum filtration through a 0.45 μm PES membrane).

The culture filtrate was concentrated using a VivaFlow 200 crossflow cassette (5000 MWCO) into 10 mm sodium phosphate buffer, pH 6.5. The concentrated protein solution was then loaded on an anion exchange column (DEAE Sepharose CL‐6B, GE Healthcare, Uppsala, Sweden), with 50 mm sodium phosphate, pH 6.5, as the loading buffer and 50 mm sodium phosphate, 1 m NaCl, pH 6.5, as the elution buffer (gradient elution: 400 mL 0–50%; 200 mL 50–100%; 200 mL 100%). SDS/PAGE was run between purification steps to track the protein. Fractions containing ~ 18 kDa large protein were pooled and selected for next purification step. The IEC was followed by size exclusion chromatography (Superdex 75 HiLoad 16/600, GE Healthcare) in 10 mm sodium acetate, pH 5.0.

Protein concentration was determined at 280 nm with a NanoDrop UV–Vis Spectrophotometer using an extinction coefficient of 0.1% = 1.71, which was calculated by Protein Identification and Analysis Tools on the Expasy Server [20]. The purified protein was concentrated to 14.12 mg·mL^−1^ in 700 μL.

### Protein crystallization

Initial screening was carried out using commercially available sparse matrix screen JCSG‐plus HT‐96 (Molecular Dimensions, Newmarket, Suffolk, UK). The following was identified as the hit condition: 12% (w/v) PEG 3350, 0.1 m HEPES, pH 7.5, 5 mm NiCl_2_, 5 mm CoCl_2_, 5 mm MgCl_2_, 5 mm CdCl_2_.

The condition was further optimized in hanging drops to 0.1 m HEPES, pH 7.5, 20% PEG 3350 and 3 mm NiCl_2_ with protein solution concentration of 14 mg·mL^−1^. Protein and reservoir proportion was 1 : 1 in hanging drops.

To reassure the identity of the crystallized protein, a protein crystal was dissolved in distilled water and the sample analyzed by SDS/PAGE (Fig. S2).

### Data collection and structure determination

X‐ray diffraction data were acquired at ID30B beamline at European Synchrotron Radiation Facility (ESRF), Grenoble, France. 7000 images were collected with an oscillation angle of 0.10° and X‐ray exposure time of 0.02 s with 4.9% transmission.

4000 images were used and processed using xds software package [21]. The structure was solved by molecular replacement with phenix.phaser from phenix suite [22] using the mature protein sequence of Cel45A from *G. trabeum* (UniProtKB: S7QB86) and coordinates from PDB entry 5KJO (*P. chrysosporium* Cel45A) as search model. The structure was refined using phenix.refine from phenix suite. Visual inspection and real space refinement were carried out using COOT [23]. Statistics are summarized in Table 1. The structure was deposited in the Protein Data Bank under ID: 8BZQ.

**Table 1:** Data collection and refinement statistics. Values taken from the validation report for the deposited structure.

Property	Value
Space group	P 1, 21, 1
Cell constants	28.97 Å, 48.41 Å, 45.79 Å
*a*, *b*, *c*, α, β, γ	90.00°, 92.21°, 90.00°
Resolution (Å)	45.76–1.30
% Data completeness (in resolution range)	99.7 (45.76–1.30)
<*I*/σ(*I*)>^1^	9.33 (at 1.30 Å)
Refinement program	phenix 1.19.2_4158
*R*, *R*_free_	0.166, 0.183
*R*_free_ test set	974 reflections (3.13%)
Wilson B‐factor (Å^2^)	9.6
Anisotropy	0.149
Bulk solvent *ksol* (e/Å^3^), *Bsol* (Å^2^)	0.34, 38.7
*L*‐test for twinning^2^	<|*L*|> = 0.49, <*L* ^2^> = 0.32
Estimated twinning fraction	0.035 for h, ‐k, ‐l
*F*_o_, *F*_c_ correlation	0.96
Total number of atoms	1481
Average B, all atoms (Å^2^)	13.0

Protein structure figures were prepared with pymol Molecular Graphics System, Version 2.0 Schrödinger (LLC, New York, NY, USA). Polar contacts were identified in pymol. Secondary structure plot was created with EMBL‐EBI online service PDBSum, where the secondary structure motifs are computed by v.3.0 of Gail Hutchinson's promotif program [24].

### Bioinformatic analysis

The protein–protein BLAST algorithm of the blastp suite was used to search for similar sequences [25, 26, 27]. The following sequences were used for query: *Mytilus edulis* MeCel45A, *Ampullaria crossean* AcCel45A, *Humicola insolens* HiCel45A, *P. chrysosporium Pc*Cel45A, *Trichoderma reesei* TrCel45A. The top 100 sequences with minimum 50% identity were collected and later revised to remove incomplete or identical sequences.

For each subfamily, a multiple sequence alignment was generated by muscle (version 5) tool for multiple alignment [28]. Consensus sequences were created using ugene (version 41.0) [29, 30, 31], with consensus type “strict” and 90% threshold. Minimum of 50 sequences were used for the generation of each consensus sequence. Visualization of consensus sequence alignment was carried out using espript 3.0 [32]. To determine the percentage of sequence identity between *Gt*Cel45A and *Pc*Cel45A, and Cel45A from *Gymnopilus dilepis*, pairwise sequence alignments were made using EMBOSS Needle available online at the EMBL‐EBI server (ebi.ac.uk) [33]. t‐coffee (Version 11.00) was used to create a structure‐based multiple sequence alignment [34]. Domain identification in Cel45A from *G. dilepis* was carried out with ScanProsite tool available online at Swiss Bioinformatics Resource Portal (expasy.org) [35].

To mark the secondary sequence elements, the following crystal structures were visualized in pymol and used as a template for the corresponding subfamily: HiCel45A (PDB ID: 2ENG), MeCel45A (PDB ID: 1WC2), *Gt*Cel45A (PDB ID: 8BZQ), and a homology model for TrCel45A. A homology model for GdCel45A (GenBank: PPQ98991.1) was created using RoseTTAFold [36]. pymol was used to generate the 3D structure images of all GH45 enzymes in this paper. All figures were made ready for publication using a vector design application affinity designer (version 1.10, 8ab56).

For the modeling of cellulose chain binding in *Gt*Cel45A, *Gt*Cel45A structure was superposed with the structure of *Pc*Cel45A in complex with two cellopentaose molecules, binding in −5 to −1 and +1 to +5, respectively (PDB: 3X2M). To improve cellulose (G10) fit in the substrate binding area, adjustments were made using Coot. The glucose residue in the −1 subsite was replaced with a distorted −1 Glc from an MD simulation model of cello‐oligo binding in HiCel45A [37]. The Asn95 sidechain clashed with the +1 Glc unit and was therefore rotated to the orientation that the corresponding residue has in the *Pc*Cel45A complex structure (PDB: 3X2M). The G10 chain was merged with the *Gt*Cel45A structure regularized in Coot. The resulting structure was refined in phenix. The G10 chain was reconstructed by merging the −5 to +1 Glc units from the refined structure (BGC210‐205; including glycosidic oxygen O4 from BGC204) with the +2 to +5 units from the *Pc*Cel45A complex (BGC204‐GLC201).

### Enzyme activity assays

*Gt*Cel45A and *Pc*Cel45A at 0.1 μm concentration were each incubated at 30 °C, 400 rpm, in 0.1 m sodium acetate buffer pH 5.0 with 0.1% barley beta‐glucan (purity ~ 95%, Megazyme, Wicklow, Ireland) dissolved in water. 1 m sodium hydroxide solution was added in a 1 : 1 ratio to the samples to stop the reaction, which was followed by cooling the samples on ice.

Reducing sugar formation was determined colorimetrically by adding p‐hydroxybenzoic acid hydrazide (PHBAH, Sigma) solution: 0.1 m PHBAH; 0.2 m sodium potassium tartrate; 0.5 m sodium hydroxide solution [38]. The PHBAH solution was prepared directly before use and added in 1 : 1 ratio to the samples. Samples containing the PHBAH reagent were immediately incubated at 95 °C for 15 min and then cooled on ice for 10 min. Samples were kept at room temperature for 5 min before absorption was read at 410 nm.

## Results and Discussion

### Overall structure of GtCel45A

The final crystal structure model of *Gt*Cel45A was refined at 1.3 Å resolution and *R*/*R*_free_ values 0.166/0.183. The space group was P21 with 1 protein chain per asymmetric unit. Data collection and refinement statistics are summarized in Table 1. The structure has been deposited in the Protein Data Bank under PDB ID: 8BZQ.

*Gt*Cel45A has a globular structure, with dimensions approximating to 26 Å × 37 Å × 48 Å. It contains a six‐stranded beta barrel with a double‐psi beta barrel (DPBB) fold, also known as the N‐terminal domain of expansins, surrounded by six short helices and seven long loops. An open substrate binding groove spans across the surface and is 48 Å in length, with the widest part of around 14 Å near the center and depth around 12 Å (Fig. S1). The first four amino acids that are present in the mature protein sequence (LEER) are not visible in the crystal structure.

**Fig. 1:**
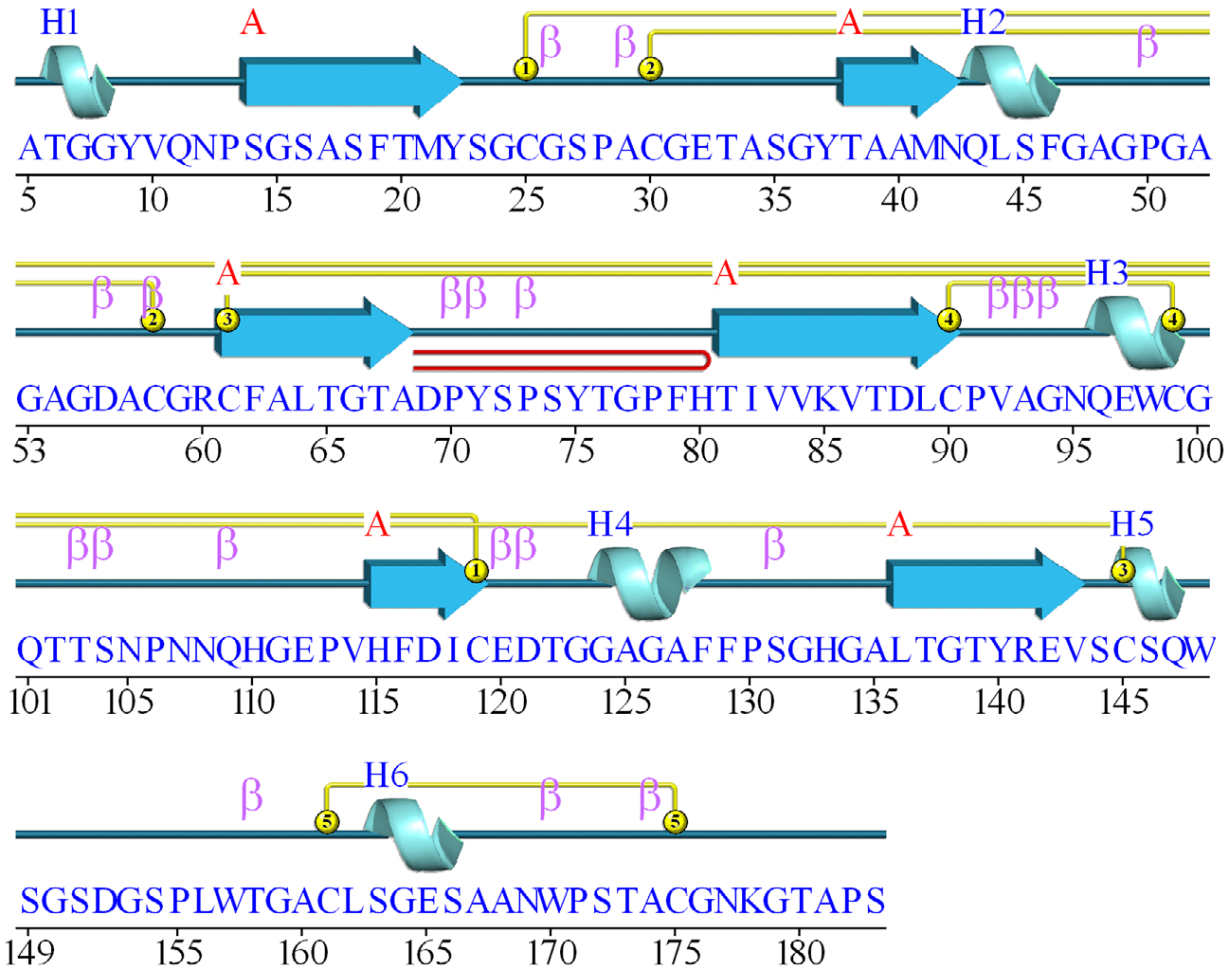
Secondary structure plot for GtCel45A. Arrows represent beta strands (A), spirals—alpha helices (H), number connections show cysteine disulfide pairings, letter β indicates a beta turn motif, a beta hairpin motif is depicted in red.

**Fig. 2:**
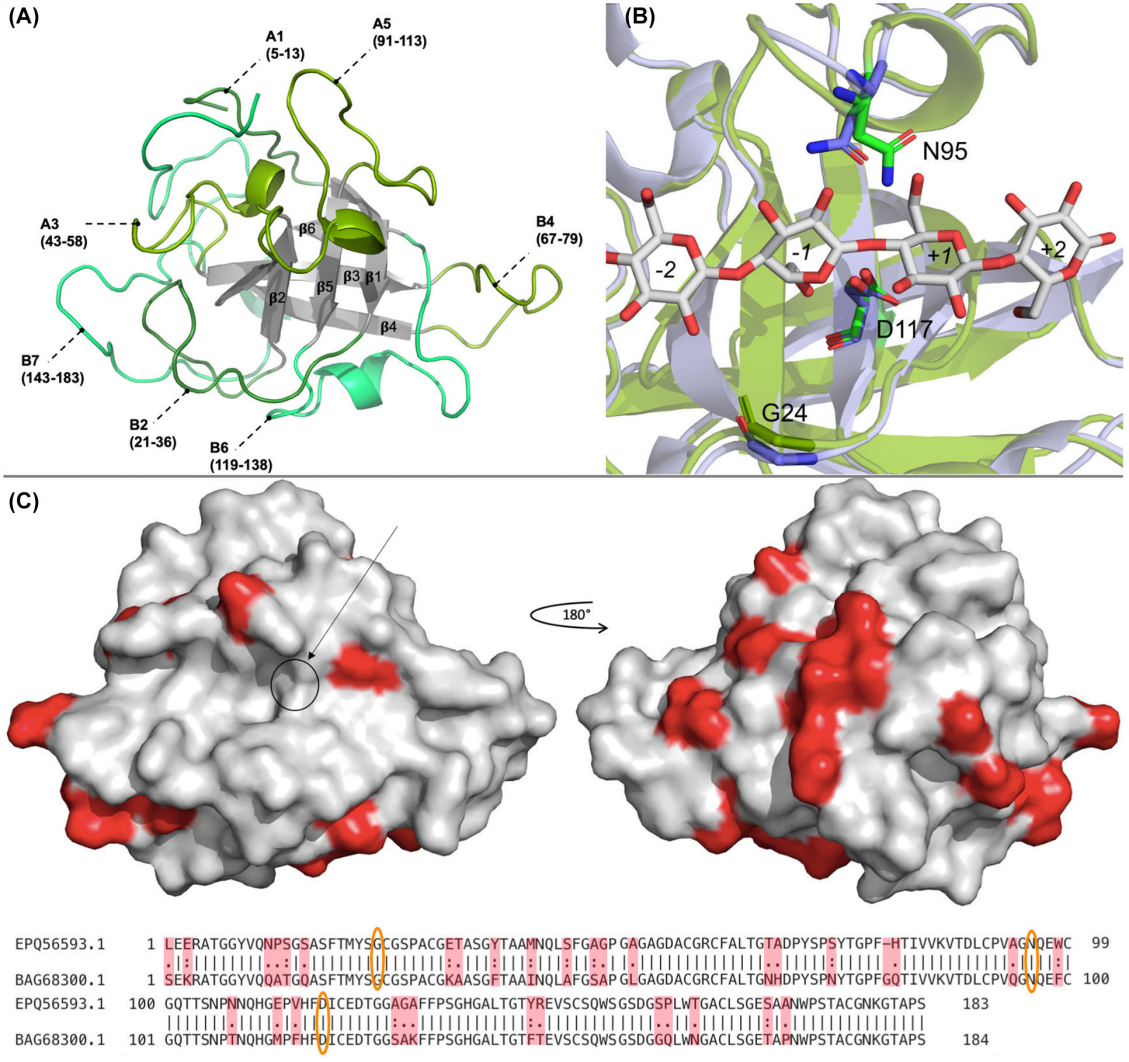
Overall structure of GtCel45A. (A) Ribbon drawing with numbering of loops, corresponding amino acid range indicated in brackets; (B) Superposed active site of GtCel45A (green) and PcCel45A (blue) with substrate bound; (C) GtCel45A surface structure. Residues in GtCel45A that are unidentical to residues in PcCel45A are indicated in red on the surface structure of GtCel45A. Catalytic center indicated with an arrow. Residues positioned at the location of catalytic residues in subfamily A are circled in orange in the sequence alignment. [Correction added on 08 February, 2024, after first online publication: The residue numbering of loop A4 in Figure 2A has been corrected in this version.]

The secondary structure along the protein sequence is depicted in Fig. 1 and the 3D structure with labeled secondary structure elements is shown in Fig. 2A.

In the following, beta strands are assigned letter β and numbered based on the order in the amino acid sequence. Loops in the structure are assigned a letter depending on their position relative to the substrate binding area (Fig. 2A). Loops positioned above the substrate binding area (where reducing end of the cellulose chain is to the right side relative to the active site) are assigned capital letter A, loops below – capital letter B. Loop numbering is based on their order in the amino acid sequence.

The structure model begins with loop A1, which contains a three amino acid long alpha helix. Five amino acids connect the helix to the first beta strand (β1), which is connected to the next beta strand (β2) via 15 amino acid loop (B2) (Fig. 2A). The secondary structure pattern of *helix –* β*‐strand –* β*‐strand* is repeated once more (Fig. 1), where at least 12 amino acid long loops connect the secondary structure elements. This pattern is followed by two repetitions of *helix –* β*‐strands*. Two helices and long loops make up the C‐terminal part of the structure.

On loop B2, shortly after strand β1, is located Tyr22 – a residue conserved in GH45 enzymes, as well as expansins. Gly24 on loop B2 is at the same position as the catalytic base in GH45 subfamily A and B members, for example Asp10 in HiCel45A and Asp24 in MeCel45A. On loop 5 (A5), between strands β4 and β5, is located an Asparagine (Asn95) residue (Fig. 2A). An asparagine in this position is regarded as the assisting residue in subfamily A enzymes. The putative catalytic acid Asp117 is found at the end of β5.

The *Gt*Cel45A structure contains five disulfide bridges formed by cysteine pairing. All disulfide bonds appear to become reduced with the increase of X‐ray exposure time during data collection. Two of the cysteine pairs (Cys61/Cys145, Cys161/Cys175) are found at either end of loop B7. Loop B7 carries Trp157 in the same position as Trp154 in *Pc*Cel45A (structure with two cellopentaose molecules, PDB ID: 3x2m) and similar to Trp64 in MeCel45A, which function as sugar‐binding platform in subsite −4.

The position of loop A5, which carries the putative alternate base Asn95, is fastened by Cys90/Cys99, which positions the Asn95 toward the catalytic acid Asp117. Molecular dynamics simulations have shown that in the absence of a substrate, a corresponding loop in *Pc*Cel45A is able to enclose toward Asp117 (Asp114 in *Pc*Cel45A), forming a polar contact between Asn95 and Asp117 (Asn92 and Asp114 in *Pc*Cel45A) [39].

The following residues have poor electron density indicated by relatively low real‐space correlation coefficient (CC < 0.9) and relatively high isotropic temperature factors (B‐factors; B_iso > 18.0): Pro50, Ala93, Gly94, Asn105, Pro130, Ser131, Gly132, His133, Thr148, Pro171, Ser172, Thr173, Thr180, Ser183. The low CC and high B factors for residues on the C‐terminal loops B6 and B7, specifically residues 130–133 and 171–183, possibly indicate dynamic disorder thus flexibility of loops B6 and B7 [40]. The electron density around Ser16 suggests static disorder with alternate conformation, assigned occupancies 0.7 and 0.3.

### GH45 subfamily C

The other molecular structure which has been deposited in PDB and belongs to subfamily C is *Pc*Cel45A. *Gt*Cel45A has 82% sequence identity with *Pc*Cel45A and 88% sequence similarity. The crystal structure of *Gt*Cel45A consists of 179 amino acids while *Pc*Cel45A – of 180. At the end of the loop B4, *Gt*Cel45A has His80, while two residues, Gly76 and Gln77, are located at this position in *Pc*Cel45A.

A total of 31 residues differ in the crystal structure of *Gt*Cel45A relative to the crystal structure of *Pc*Cel45A. Most are away from the substrate binding groove (Fig. 2C), three can be found in its periphery (Ala93/Gln90, Trp98/Phe95, Thr158/Asn155 in *Gt*Cel45A/*Pc*Cel45A respectively), but none at the catalytic site. All available *Pc*Cel45A crystal structures to date are missing the first four N‐terminal residues present in the mature protein sequence.

The subfamily C enzymes are unique in GH45 family due to the ability to achieve hydrolysis despite lacking a traditional catalytic base residue in the active site (Fig. 2B). Both subfamily A and subfamily B enzymes have aspartic acid residues acting as a catalytic base in the active site, while subfamily C has a glycine residue at this position.

**Fig. 3:**
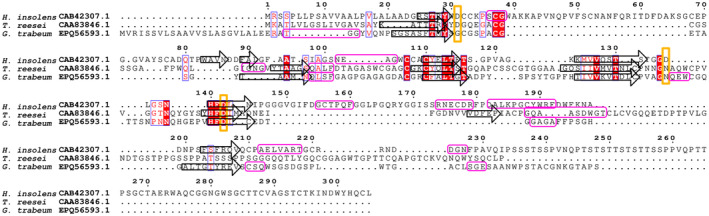
Structure‐based sequence alignment of H. insolens Cel45A, T. reesei Cel45A and G. trabeum Cel45A. Alignment visualized in espript 3.0. Secondary structure elements are represented as rounded rectangles (helices) and arrows (β‐strands). Character coloration according to ESPript 3.0: filled red box and a white character indicate strict identity; red character—similarity within a group. Active site residues of subfamily A and residues at the corresponding location in other subfamilies are marked with a yellow frame.

It has been suggested that subfamily C members utilize an asparagine residue (Asn95 in *Gt*Cel45A) in their catalytic mechanism, instead of using the residue corresponding to the position of catalytic base in the other two subfamilies. The Asn95 is positioned on a loop enclosing toward the active site. Asparagines are conserved at this location in subfamily B and C, and aspartic acid—in subfamily A (Fig. 3). Mutation of the asparagine residue at this position in the subfamily C enzymes from *P. chrysosporium* and *Fomitopsis palustris* leads to a significant decrease in their hydrolytic activity [41].

The catalytic mechanism attributed to the hydrolytic properties of subfamily C is the “Newton's cradle” proton relay mechanism which originally was proposed in *Pc*Cel45A. This mechanism involves a proton relay through hydrogen bonding network from the catalytic acid to the proposed catalytic base. In *Pc*Cel45A, it involves 9 amino acids: Asn92, Gln93, Cys96, Phe95, Asn105, Ser14, His112, Thr16, Asp114 [18]. *Gt*Cel45A has conserved most of the amino acids involved in the “Newton's cradle” proton relay mechanism (Asn95, Gln96, Cys99, Trp98, Asn108, Ser18, His115, Thr20, Asp117), except for residue Phe95 (Trp98 instead). According to this mechanism, the imidic acid form of Asn95 could act as a general base in *Gt*Cel45A.

MD simulations [39] with celloheptaose molecule bound in the substrate‐binding groove have shown that residues interacting with substrate the strongest in *Pc*Cel45A are Asp85 (subsite −2), Asn92 (subsite −1), Trp154 (subsite −4). The corresponding residues in *Gt*Cel45A (Asp88, Asn95, Trp157) are located in the same positions. Other residues assumed to interact with celloheptaose in *Pc*Cel45A (Gly47, Leu86, Tyr18, Asp114, Thr16, Met17, His107, Gly131, Tyr67) are also the same in *Gt*Cel45A (Gly51, Leu89, Tyr22, Asp117, Thr20, Met21, His110, Gly134, Tyr71).

Consensus sequences show that 14 residues are strictly conserved in all of the GH45 subfamilies known to date (Fig. S3). Subfamily B sequences appear less conserved, presumably due to the larger diversity of organisms expressing subfamily B enzymes – mollusks and fungi.

As mentioned previously, the absence of an acidic residue at the catalytic base position distinguishes subfamily C from the other two subfamilies (Fig. S3). However, there is further diversity among these enzymes which requires investigation. Most subfamily C enzymes are one domain proteins, but at least one appears to have a CBM, *Gymnopilus dilepsis* Cel45A (GenBank: PPQ98991.1) [42], which has an N‐terminal CBM1 domain connected by a linker to the catalytic domain (Fig. S4). After removal of the CBM1 and linker from the sequence, *G. dilepis* Cel45A (*Gd*Cel45A) becomes 75% sequence identical to *Gt*Cel45A.

Thus far studies regarding enzymatic activity have not been carried out at identical conditions, therefore a uniform opinion on the catalytic activities of subfamily C enzymes cannot be made. It is clear that GH45 subfamily enzymes are able to hydrolyze CMC, barley beta‐glucan and glucomannan [14, 15, 19, 41]. Studies show that the pH optimum is around pH 5, when determined on CMC, and some of GH45 subfamily C enzymes appear stable at 50 °C [19, 41].

**Fig. 4:**
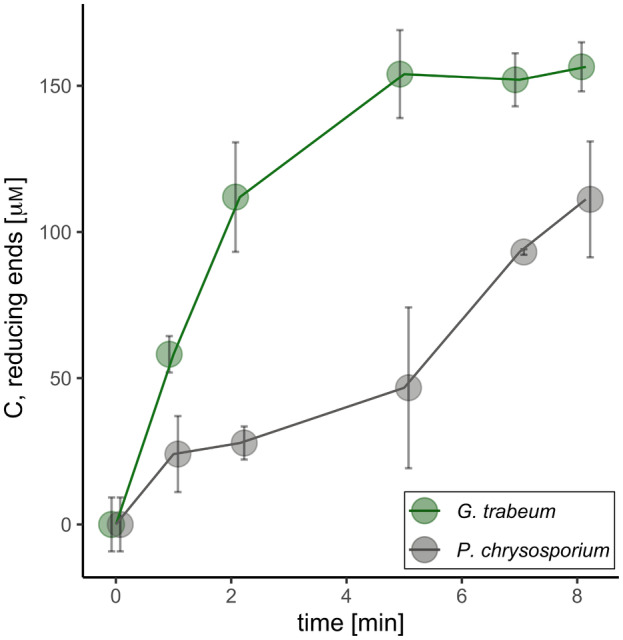
Hydrolytic activity comparison of 0.1 μm GtCel45A and 0.1 μm PcCel45A on 0.1% barley beta‐glucan, at +30 °C, pH 5.0. Error bars represent SD.

We compared the hydrolytic activity of *Gt*Cel45A and *Pc*Cel45A on barley beta‐glucan at 30 °C, pH 5. Despite the high sequence and structural similarity, *Gt*Cel45A exhibited ~ 3.8 times higher initial rate – it took 2.15 min for 0.1 μm *Gt*Cel45A to produce 112 ± 19 μm of reducing ends and 8.15 min for 0.1 μm *Pc*Cel45A to produce 111 ± 20 μm. Moreover, *Gt*Cel45A reached a plateau faster than *Pc*Cel45A (Fig. 4). At present we do not have any explanation for that difference.

## Conclusions

Cel45A from the brown‐rot fungus *G. trabeum* is a GH45 subfamily C member with a nearly identical structure to the Cel45A from white‐rot fungus *P. chrysosporium*, and yet shows higher catalytic activity. The diversity within GH45 subfamily C would benefit from elucidation, as there is minimum one subfamily C member which has a CBM1 domain, the Cel45A from *G. dilepis*. The GH45 family is strictly conserved regarding 14 residues.

## Conflict of interest

The authors declare no conflict of interest.

### Peer review

The peer review history for this article is available at https://www.webofscience.com/api/gateway/wos/peer‐review/10.1002/2211‐5463.13774.

## Author contributions

LO: Conceptualization, Validation, Methodology, Formal analysis, Investigation, Data Curation, Writing – Original Draft, Writing – Review & Editing, Visualization, Supervision. LF: Validation, Methodology, Formal analysis, Investigation, Data Curation. MS: Writing – Review & Editing, Resources, Supervision, Project administration, Funding acquisition. JS: Conceptualization, Validation, Methodology, Formal analysis, Resources, Data Curation, Writing – Original Draft, Writing – Review & Editing, Supervision, Project administration, Funding acquisition.

## Supporting information


**Fig. S1.** Measurements of GtCel45A dimensions expressed in Å and depicted as dashed lines.
**Fig. S2.** SDS/PAGE analysis of a dissolved protein crystal shows a single band at ~18 kDa, the expected molecular weight of GtCel45A, thus confirming the identity of the purified and crystallized protein.
**Fig. S3.** (A) An alignment of GH45 subfamily A, B and C consensus sequences; (B) An alignment of subfamily B consensus sequence, subfamily B consensus sequence in phylum Ascomycota, and phylum Mollusca.
**Fig. S4.** Homology structure model of the bimodular GH45 subfamily C enzyme GdCel45A from Gymnopilus dilepis with linker and CBM1.

## Data Availability

The structural data that support these findings are openly available in the wwPDB at https://doi.org/10.2210/pdb8BZQ/pdb. The data that support the findings of this study are openly available in enzyme nomenclature database – ENZYME at https://enzyme.expasy.org, Enzyme Commission number: EC3.2.1.4.
