# Correction to “The first crystal structure of a family 45 glycosidehydrolase from a brown‐rot fungus, *Gloeophyllum trabeum* GtCel45A”

**DOI:** 10.1002/2211-5463.70040

**Published:** 2025-04-21

Laura Okmane , Louise Fitkin , Mats Sandgren , Jerry Ståhlberg . The first crystal structure of a family 45 glycosidehydrolase from a brown‐rot fungus, *Gloeophyllum trabeum* GtCel45A. FEBS Openbio. 2024;14: 505‐514. 10.1002/2211-5463.13774 38311343 PMC10909974

The authors include the following sentence in the acknowledgements section:

“We acknowledge the European Synchrotron Radiation Facility for provision of synchrotron radiation facilities and we would like to thank the staff of the ESRF and EMBL Grenoble for assistance and support in using beamline ID30B.”

The acknowledgements section has been updated as:

We thank Dr. Fernando Segato, University of São Paulo, Brazil, for providing spores of the *Gt*Cel45A expressing *A. nidulans* strain. This work was supported by the Swedish Research Council for Environment, Agricultural Sciences and Spatial Planning (Formas), Grant Number 2017–01130. We acknowledge the European Synchrotron Radiation Facility for provision of synchrotron radiation facilities and we would like to thank the staff of the ESRF and EMBL Grenoble for assistance and support in using beamline ID30B.

