# Supplementary material for: The first crystal structure of a family 45 glycoside hydrolase from a brown‐rot fungus, Gloeophyllum trabeum GtCel45A
Source: FEBS Open Bio. 2024 Feb 4;14(3):505–14. doi: 10.1002/2211-5463.13774 (PMC10909974; doi:10.1002/2211-5463.13774)
Supplement: Supplementary file 1 — Fig. S1. Measurements of GtCel45A dimensions expressed in Å and depicted as dashed lines. Fig. S2. SDS/PAGE analysis of a dissolved protein crystal shows a single band at ~18 kDa, the expected molecular weight of GtCel45A, thus confirming the identity of the purified and crystallized protein. Fig. S3. (A) An alignment of GH45 subfamily A, B and C consensus sequences; (B) An alignment of subfamily B consensus sequence, subfamily B consensus sequence in phylum Ascomycota, and phylum Mollusca. Fig. S4. Homology structure model of the bimodular GH45 subfamily C enzyme GdCel45A from Gymnopilus dilepis with linker and CBM1. [file FEB4-14-505-s001.docx]

Supplementary Information

**The first crystal structure of a family 45 glycoside hydrolase from a brown-rot fungus, *Gloeophyllum trabeum* GtCel45A**

Laura Okmane, Louise Fitkin, Mats Sandgren, Jerry Ståhlberg

Department of Molecular Sciences, Swedish University of Agricultural Sciences, Uppsala, Sweden

Corresponding author: Laura Okmane, Department of Molecular Sciences, Swedish University of Agricultural Sciences, POB 7015, SE-750 07 Uppsala, Sweden. Email: Laura.Okmane@slu.se

# **Table of contents**

## Supplemental figures

Figure S1. Measurements of GtCel45A dimensions expressed in Å.

Figure S2. SDS-PAGE analysis of a dissolved protein crystal.

Figure S3. GH45 consensus sequence alignments.

Figure S4. Homology structure model of GdCel45A from *Gymnopilus dilepis*.


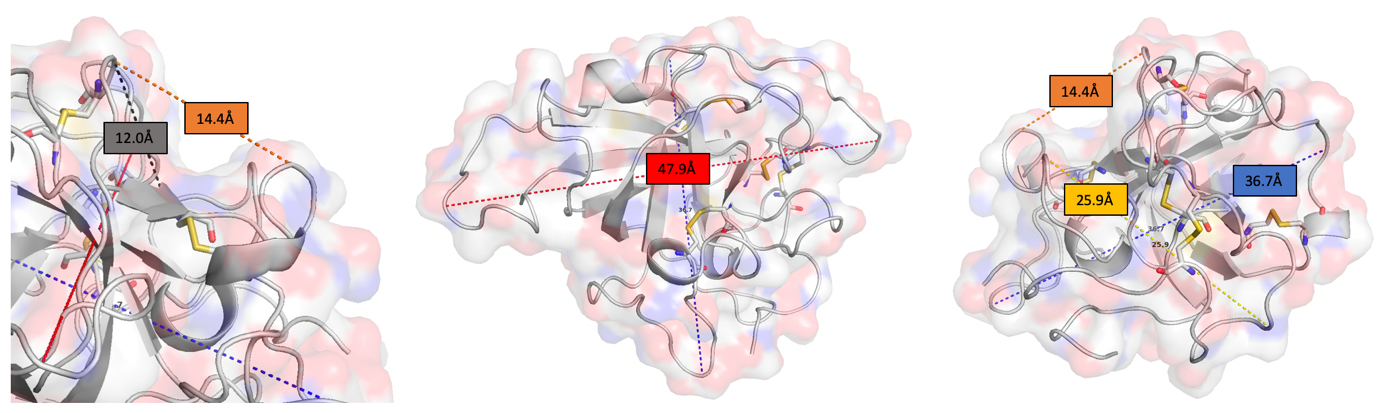


**Figure S1. Measurements of GtCel45A dimensions expressed in Å and depicted as dashed lines.** The depth and depth of middle part of the substrate binding groove indicated with dark gray and orange, respectively. The length of the substrate binding groove is indicated in red. The height and width are indicated in blue and yellow, respectively.


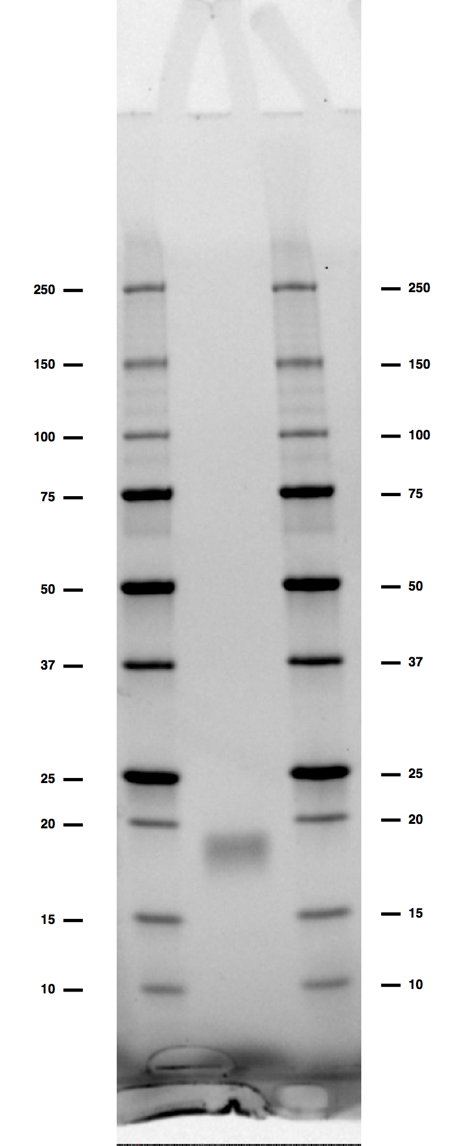


**Figure S2. SDS-PAGE analysis of a dissolved protein crystal shows a single band at ~18 kDa, the expected molecular weight of GtCel45A, thus confirming the identity of the purified and crystallized protein.** Standards for molecular weight determination are loaded in adjacent wells.

Figure S3. **(A) An alignment of GH45 subfamily A, B and C consensus sequences; (B) An alignment of subfamily B consensus sequence, subfamily B consensus sequence in phylum Ascomycota, and phylum Mollusca.** Character coloration according to ESPript 3.0: filled red box and a white character indicate strict identity; red character – similarity within a group; blue frame – similarity across groups. Residues which are not part of the consensus sequence, have been omitted. (B)

Figure S4. **Homology structure model of the bimodular GH45 subfamily C enzyme GdCel45A from *Gymnopilus dilepis* with linker and CBM1.** The surface structure and domain organization are shown in panel A, and C-terminal domain overlay with GtCel45A in panel B. Conserved catalytic site residues are indicated for GdCel45A.
